# Thrombosis of the Brachial Artery After Closed Elbow Dislocation

**DOI:** 10.7759/cureus.44627

**Published:** 2023-09-04

**Authors:** Bensitel Omar, El Fahd Othmane, El Jebbouri Khalid, Rahmi Mohammed, Rafai Mohamed

**Affiliations:** 1 Orthopedic Surgery P32, Ibn Rochd University Hospital, Casablanca, MAR; 2 Orthopedic Surgery P32, Faculty of Medicine and Pharmacy, Hassan II University of Casablanca, Casablanca, MAR

**Keywords:** complications of elbow dislocation, vascular graft, elbow injury, brachial artery thrombosis, elbow dislocation

## Abstract

Posterior dislocation of the elbow joint is the second commonest large joint dislocation that can be experienced due to various traumatic incidents. Although it may be associated with fractures and vascular lesions, in this case report, we describe a patient who encountered a posterolateral elbow dislocation following a fall on their arm with an extended elbow. This dislocation was followed by delayed thrombosis of the brachial artery, necessitating a revascularization surgery. For optimal patient care, physicians should remain vigilant, being cautious about potential vascular injuries both before and after performing a closed reduction of the elbow joint. The suspicion of vascular injury should be even more pronounced when bony lesions or open injuries are present. Effective management of such cases requires a collaborative effort between orthopedic and vascular surgeons. The preferred surgical approach involves the utilization of a saphenous graft, with the essential prerequisite of achieving a stable elbow joint before proceeding with revascularization.

## Introduction

The occurrence of brachial artery injury following a closed dislocation of the elbow is unusual, with an approximate incidence of 0.5% [1]. Brachial artery damage is predominantly linked to open elbow dislocations or penetrating wounds in the elbow area, but it is rare in patients with closed dislocations [1]. It is essential to maintain a high level of suspicion regarding this potentially severe complication adding nerve damage [2]. This report details a unique occurrence of a complete thrombosis of the brachial artery, which was concomitant with a posterior elbow dislocation after a traumatic event.

## Case presentation

**Figure 1:**
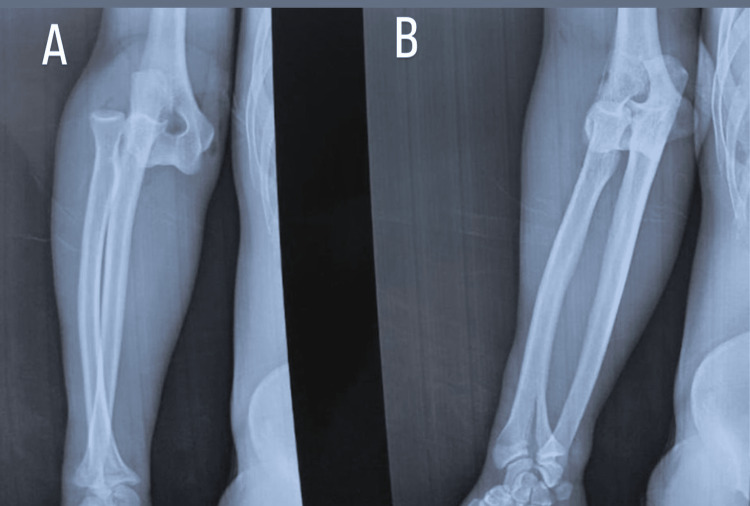
Elbow radiographs demonstrate posterior elbow dislocation before reduction, with no signs of bone lesions. (A) Anteroposterior (AP) view of elbow X-ray. (B) Lateral view of elbow X-ray

**Figure 2:**
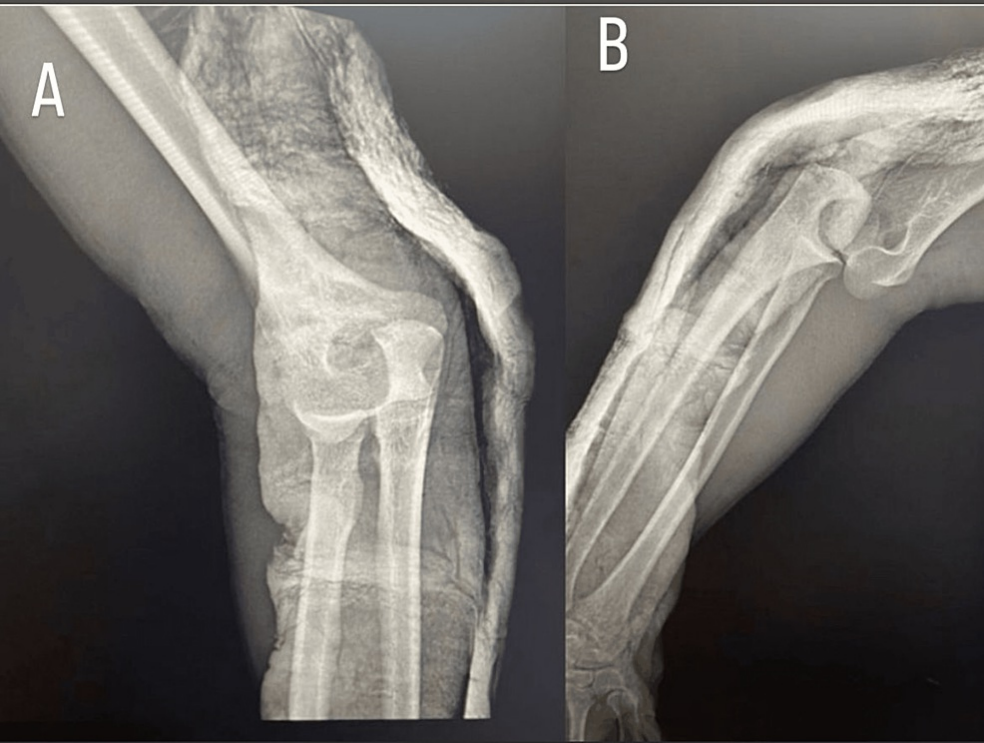
Radiographic view after closed reduction, where joint congruence is restored. (A) Anteroposterior (AP) view of elbow X-ray. (B) Lateral view of elbow X-ray

A 34-year-old right-hand-dominant male was admitted to our Emergency Department after experiencing a fall from a standing height during a football match, landing on his left arm. Medical history is insignificant. Upon presentation, he showed evident elbow deformity, swelling, and persistent tingling sensations in his hand. No associated wounds were observed. Despite the normal limb temperature and the capillary refill time under three seconds, radial and ulnar pulses could not be palpated at the wrist. X-rays confirmed a closed posterior dislocation of the elbow without any associated fractures (Figure 1). While the patient was under monitored sedation, a successful immediate closed reduction was performed. This process entailed gripping the patient's wrist, ensuring it remained supinated, applying axial traction, and flexing the elbow to maintain relaxation in the triceps muscles. As a result, this procedure not only restored the alignment of the joint but also alleviated hand paresthesia (Figure 2). However, the radial and ulnar pulses could not be appreciated by palpation. A color Doppler ultrasound was prescribed. It confirmed a brachial artery thrombosis complicating subacute ischemia of the forearm. The decided treatment was concomitant between the orthopedic and vascular surgeons. Urgent surgical exploration was carried out to address the situation one hour after the patient’s admission.

**Figure 3:**
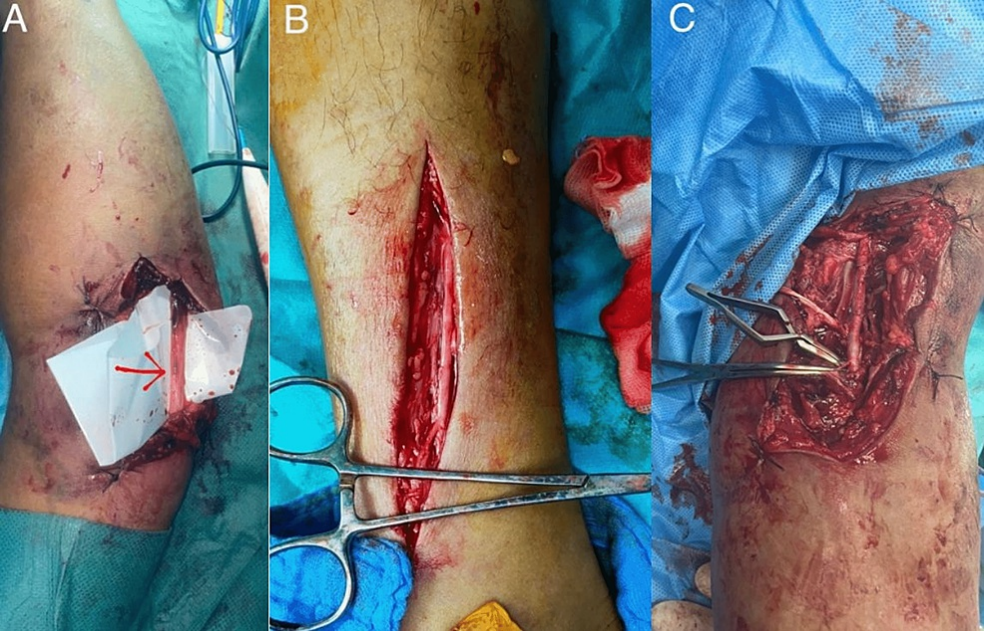
Interposition of a saphenous vein graft for brachial artery repair. (A)  Thrombosed brachial artery along a length of 4 centimeters. (B and C) The interposition graft utilized the distal portion of the ipsilateral saphenous vein.

During the intervention, the brachial artery was found to be inflamed, scarred, and thrombosed along a length of 4 cm (Figure 3). A 5 cm interposition graft, using the ipsilateral saphenous vein, was performed with complete restoration of the distal radial and ulnar pulses. The stability of the elbow joint was thoroughly assessed after the surgical procedure, revealing no indications of instability. However, it was determined that a fixed 90-degree elbow splint would be utilized for three weeks to protect the neo-vascular graft. A postoperative CT scan was conducted to rule out any concealed bone avulsions, and no complications arose during the recovery period. Following consultation with a vascular surgeon, a decision was made to pursue non-surgical management for the patient. Subcutaneous enoxaparin was initiated and administered for a week, followed by lifelong daily intake of aspirin (150mg). Histopathological examination showed no sign of an intimal flap. The patient was discharged six days after the surgery, and upon removing the splint, they began a specific rehabilitation program.

During the one-month follow-up visit, the patient exhibited pink fingers with a capillary refill time of less than three seconds. Sensory and motor functions in the fingers were intact. However, distal pulses remained unpalpable. After the elbow slab was taken off, the range of motion was found to be within the correct parameters.

During the three-month follow-up examination, the patient exhibited a range of motion in the affected limb that was within normal parameters when compared to the contralateral limb. The patient did not report any pain or cold intolerance. Palpation of radial and ulnar pulses indicated slightly weaker pulses on the affected side compared to the contralateral side. No signs of Volkmann's ischemic contracture were observed.

## Discussion

The elbow joint benefits from a robust collateral arterial blood supply. The ulnar artery forms connections through the superior and inferior collateral ulnar arteries. Likewise, the proximal portion of the brachial artery is linked to the radial artery by the anterior branch of the deep brachial artery [3].

In cases of blunt trauma to the elbow accompanied by indications of fracture or dislocation, healthcare providers must be vigilant by maintaining a high level of suspicion for potential brachial artery injury. Detectable signs of limb ischemia might not be immediately evident due to the ample collateral blood supply in the upper limb. Instead, patients may present with mild indicators, such as palpable but weakened pulses and delayed capillary refill [4]. In cases where closed dislocations coexist with preserved neurovascular function and arterial injuries, about 10% of individuals may display palpable pulses distal to the trauma site [5]. For instance, in a documented case by Eijer et al., an arteriogram was performed on a patient who displayed a strong radial pulse, revealing occlusion of the brachial artery [6].

CT angiography is considered the gold standard for diagnosing arterial injuries. However, some proponents of Doppler ultrasonography argue that it offers advantages such as bedside convenience and less invasiveness. On the other hand, researchers like Marcheix et al. do not recommend its use, citing reasons such as operator dependency and challenges in performing the procedure on an injured elbow [7]. In a well-documented case, Masionis et al. emphasize the importance of angiography in differentiating between thrombosis and rupture of the brachial trunk. If rupture is evident, the existence of swelling and a significant hematoma could potentially interfere with the flow of collateral blood supply [8].

In cases where the neurovascular status is preserved, prioritizing surgical treatment involving either a saphenous vein graft or direct suture is essential to prevent future complications [9]. Many authors suggest utilizing a saphenous vein graft as the initial treatment option, as its length and diameter can readily accommodate the specific requirements of the situation [10]. Our analysis of this case and the literature highlights the importance of a collaborative approach involving both a vascular surgeon and a trauma surgeon in managing this condition. Successful repair of vascular damage hinges on achieving a stable elbow joint. The trauma surgeon, with the assistance of the vascular surgeon, should thoroughly examine the elbow joint before and after closed reduction particularly when dealing with the presence of neurological deficits (particularly median nerve palsy), or significant swelling around the elbow [11]. Ultimately, the most suitable course of action should be determined by the trauma surgeon.

## Conclusions

Brachial artery thrombosis following posterolateral dislocation can have severe consequences. Physicians should maintain a high level of suspicion for concurrent vascular injuries both before and after closed reduction of the elbow joint. This underscores the necessity for constant attentiveness and meticulous follow-up to avoid the potentially catastrophic complication of an ischemic arm. Collaboration between trauma and vascular surgeons is crucial, as surgical treatment using a saphenous graft is the preferred approach, provided that the elbow joint stability is ensured.
